# 

**Published:** 1893-06

**Authors:** 


					CONTENTS.
^S"lfn?Pia and Ocular Headache.
MyBF'LondCR!ss' F:.R-C-S:.Eng:;
73
Thrombosis of the Femoral
ARTHrLL|a ning t0 Gangrene^
By
Arthur E. Barker, F.R.C.S. Eng.
81
W?n5^?ea Branchial Fistula.
Cantab?RGE Parker- M'D"' M,a"
89
'I ?neYi,
*iew of Heredity.
By Augustin
BerHnARD' F"R-C-S- Eng-. M.D.
Heap?h: Resets in the West of
T??land a"d South Wales,
??rquay.
By Paul Q. Karkeek,
W-R.C.S. Eng., L.S.A."' ... '
Progress of the Medical Sciences:
Medicine.
By R. Shingleton
Smith, M.D....
100
Surgery.
By W. H. Harsant
104
Obstetrics.
By L. M. Griffiths ...
108
Reviews of Books
113
Notes on Preparations for the Sick
132
Meetings of the Bristol Medico-
Chirurgical Society
135
The Library of the Bristol Medico-
Chirurgical Society
137
The Hodgkins Fund Prizes
141
The Bristol Eye Hospital
142
Scraps
143

				

